# Investigating the satisfaction level of physicians in regards to implementing medical Picture Archiving and Communication System (PACS)

**DOI:** 10.1186/s12911-020-01203-0

**Published:** 2020-08-05

**Authors:** Reza Abbasi, Monireh Sadeqi Jabali, Reza Khajouei, Hamidreza Tadayon

**Affiliations:** 1 Student Research Committee, Kerman University of Medical Sciences, Kerman, Iran; 2 Research Centre for Health Information Management, Kashan University of Medical Sciences, Kashan, Iran; 3 Medical Informatics Research Center, Institute for Futures Studies in Health, Kerman University of Medical Sciences, Kerman, Iran; 4 Faculty Member of Health Information Technology Department, Neyshabur University of Medical Sciences, Neyshabur, Iran

**Keywords:** Picture Archiving and Communication System (PACS), Satisfaction, Usability, Health information systems

## Abstract

**Background:**

User satisfaction with PACS is considered as one of the important criteria for assessing success in using PACS. The objective of this study was to determine the level of user satisfaction with PACS and to compare its functional features with traditional film-based systems.

**Methods:**

This study was conducted in 2017. Residents at three large university hospitals in Kerman filled-out a self-administered questionnaire consisting of three parts: demographic information of participants, user satisfaction with PACS, comparing features of the two digital and traditional imaging systems. The validity of this questionnaire was approved by five medical informatics, radiology, and health information management specialists and its reliability was confirmed by Cronbach’s alpha (86%). Data were analyzed using descriptive statistics and the Spearman, Mann Whitney U and Kruskal-Wallis statistical tests.

**Results:**

The mean of the participants’ ages was 31.4 (±4.4) years and 59% of the participants were females. The mean of physicians’ satisfaction with PACS’ had no significant relationship with their age (*P* = 0.611), experience of using PACS (*P* = 0.301), specialty (*P* = 0.093), and percent of interpretation of images with PACS (*P* = 0.762). It had a significant relationship with the participants’ computer skills (*P* = 0.022).

**Conclusions:**

The mean of physicians’ satisfaction with PACS was at a moderate to a high level, yet there are still problems in the successful implementation of these systems and establishing interoperability between them**.** PACS has not fully met all the demands of physicians and has not achieved its predetermined objectives, such as all-access from different locations**.**

## Background

Using modern technologies has led to the optimization of the quality and productivity of the health care systems [1, 2]. Picture Archiving and Communication System (PACS) is one of these modern technologies. Due to the high costs of radiology films and their storage, their retrieval problems, and problems with the distribution and display of traditional radiology film-based pictures [3, 4], PACS has become the heart of the medical imaging centers [5].

PACS, as a module integrated with the radiology information system, is a centralized source for all imaging data that creates and transfers digital radiology pictures and their reports [6]. This system helps users to change picture display parameters such as quality, zoom, and contrast and to compare pictures through a workstation computer [7, 8]. PACS has become an alternative for traditional film-based imaging since the 1980’s [1] due to many advantages such as optimizing image quality and their accessibility [3, 7, 9, 10], increasing physicians’ productivity and efficiency [3, 9], improving the connection between clinical units and radiology department [7], reducing the number of lost images [3], decreasing the time of reports and sending radiology reports [3, 11, 12], lowering the need to physical space for picture archiving [13], reducing personnel costs and expenses related to films and relevant chemical substances [4, 12, 14], decreasing the need for re-imaging and patients’ exposure to harmful rays [3, 14, 15], and reducing the average waiting time for patients [9, 16, 17]

Despite the crucial role and advantages of PACS for healthcare centers the implementation and use of these systems have faced some challenges [16]. Therefore the successful implementation of this system should be seriously addressed [18]. User satisfaction is one of the factors affecting successful implementation of information systems [19–21]. So that user resistance towards using these systems leads to inefficiency [22] and eventually results in system withdrawal [23, 24]. Since user satisfaction with an information system leads to employees’ productivity [25] and their continuous use of this system [19] identifying and resolving issues provoking users’ dissatisfaction seems necessary. Many studies have investigated users’ satisfaction with PACS [10, 15, 17, 26, 27] and reported different findings. The results of one study revealed that only 8% of radiologists believed that using PACS was easier than film-based imaging [17]. However another study showed users’ satisfaction with the quality of information and images produced by PACS and shed a light on using this technology compared to the traditional systems [10]

During recent years, several Iranian hospitals have taken action to implement and use PACS. Iranian Ministry of Health has approved 5 PACSs, which are accordant with the DICOM standard, to be used in healthcare organizations. The systems being studied in the present research have different capabilities including, measurement of lesions, ability to measure Hounsfield units, ability to make visual changes such as brightness, contrast, images resolution, and zooming in and out, viewing images in different slices, archiving images on database or CD, communicating images with workstations at the centers and ability to retrieve images on workstations. There are also studies concerning the challenges and issues in implementing PACS [28, 29], factors affecting its use, successes and failures of this system [16, 30, 31], evaluation of usability [32, 33] and investigating the impact of its implementation [11]. However, according to our knowledge, no study has been conducted in Iran to assess physician satisfaction with PACS. Since, physicians are one of the primary groups of PACS users, this study was conducted to determine the physicians’ satisfaction with PACS and to compare this the functional features of this system to the traditional film-based imaging system.

## Methods

### Research setting

The present study was conducted in 2017. All available senior residents (*n* = 59) who had the experience of using PACS at three large university hospitals in Kerman were included in this study. Kerman University of Medical Sciences is the largest medical university in southeast Iran that has three large general hospitals (Afzalipour, Shafa, and Bahonar), with 462, 420 and 252 active inpatient beds; respectively. These hospitals have various inpatient wards such as emergency, orthopedic, internal medicine, neurology, pediatrics, obstetrics and gynecology, rheumatology, infectious, ENT, dermatology, nephrology, cardiology, endocrinology, dialysis, burn, general surgery, ICU, CCU.

### Data collection

Data were collected using a self-administered questionnaire that was developed by the researchers based on the review of relevant studies [1, 9, 15, 34]. This questionnaire consisted of three parts;

The first part included questions related to demographic information including age, gender, specialty, experience of using PACS, computer skills (self-report) and also the percent of images interpreted with PACS by physicians.

The second part included 14 questions regarding the individuals’ satisfaction with the PACS using a 7-choice Likert scale (from “completely disagree” to “completely agree”).

3) The third part included 8 questions related to comparing features of the two digital and traditional film-based imaging systems using a 5-choice Likert scale (from score 1 to 5).

Also, to collect other positive and negative aspects of the PACSs, two open questions were added at the end of the questionnaire.

The face and content validity of this questionnaire was reviewed and approved by five medical informatics (*n* = 2), radiology (*n* = 2), and health information management (*n* = 1) specialists. These specialists were faculty members in the two medical sciences universities with at least 6 years of work experience. The mean and SD of their age was 42.6 ± 5.2. The questionnaire was filled out by 12 random residents, and its reliability was confirmed using Cronbach’s alpha (α = 0.86). To collect the data, the researchers visited all three hospitals and, after explaining the aim of the study, distributed the questionnaires amongst physicians who had consented to take part in the study.

### Data analysis

Data were analyzed using SPSS.18. After checking the normality of the data, the Spearman statistical tests were used to assess the relationship between the mean score of the overall satisfaction with PACS and users age and experience of using the PACS; Mann Whitney U was used to investigate its relationship with individuals’ gender; and Kruskal-Wallis test was used to determine its relationship with the users’ specialty, percent of images interpreted with PACS and computer skills. Physicians’ satisfaction with PACS was evaluated using the second part of the questionnaire. We applied the method used in a previous study [35] to categorized the overall satisfaction of the participants. Hence, based on the minimum and maximum attainable scores in this questionnaire (14 and 98), we classified the overall satisfaction with PACS into three categories: low (14–42), moderate (43–70), and high (71–98).

## Results

Table 1 shows demographic information and the mean scores of physicians’ satisfaction with PACS. Forty-six physicians participated in this study. The mean age of the participants was 31.4 (± 4.4) years and approximately 59% (*n* = 27) of the participants were females. About 30% (*n* = 14) of the participants were emergency medicine residents, 26% (*n* = 12) internal residents and 20% (*n* = 9) radiology residents. Almost 67% (*n* = 31) of the physicians stated having an intermediate level of computer skills. The mean score of experience with PACS was (13.3 ± 10.5) months. About 77% (*n* = 35) of the physicians stated that they interpret more than half of the medical images using PACS. Results of this study showed that the mean score of physicians’ satisfaction with PACS’ has no significant relationship (*P* > 0.05) with participants’ age, experience of using PACS, type of specialty, percent of images interpreted with PACS. However, it had a significant relationship with their computer skills (*P* < 0.05).

**Table 1:** The mean scores of participants’ satisfaction based on their demographic information

Demographic information	Frequency (%)	Mean score of satisfaction	*p*-value
Gender^a^			
Female	27(59)	4.02 ± 1.61	0.126
Male	19(41)	4.65 ± 1.86	
Specialty^b^			
Emergency Medicine	14(30)	4.09 ± 1.9	0.093
Internal Medicine	12(26)	3.35 ± 1.73	
Radiology	9(20)	5.71 ± 1.07	
Orthopedics	4(9)	4.28 ± 1.62	
Cardiology	3(7)	4.09 ± 1.13	
Pediatrics	2(4)	3.78 ± 1.01	
Urology	1(2)	4.85	
Neurology	1(2)	6.14	
Computer skills^b^			
Low	7(15)	3.38 ± 1.92	0.022
Intermediate	31(67)	4.13 ± 1.7	
High	8(18)	5.62 ± 0.45	
Percent of images interpreted with PACS^b^			
(1–25) %	1(2)	4.57	0.762
(26–50) %	9(20)	3.6 ± 1.42	
(51–75) %	14(31)	4.71 ± 1.09	
(76–<100) %	12(26)	4.39 ± 2.11	
100%	9(20)	4.15 ± 2.4	

^a^ Mann-Whitney U

^b^Kruskal-Wallis

Approximately 58% of the physicians agreed that using PACS is a great achievement for their hospitals. Also, 50% of the participants believed that using PACS reduces image interpretation time, and 59% of them also stated that reviewing images with this system is easy. About 76% of physicians believed that the quality of PACS images is higher than radiography films. Over 60% of the physician agreed that PACS leads to less time in searching for images, accelerates diagnosis time, and reduces any ambiguity of the images. Also, the same number of physicians believed that using this system optimizes in the work process and training. About 56% of physicians believed that PACS has improved the quality of care. Around 52% of the participants expressed that PACS does not reduce patients’ admission time in the hospital. Over 50% of the physicians also said that PACS reduces the costs and meets the users’ expectations.

As displayed in Table 2, the satisfaction level of 41% (*n* = 19) of the physicians with the PACS was at a high level and overall, the satisfaction level of 72% of the physicians was moderate to high.

**Table 2:** Overall satisfaction level with PACS

	Satisfaction Level	Frequency (%)
Overall Satisfaction with PACS	Low	13 (28)
	Moderate	14 (31)
	High	19 (41)

The mean satisfaction scores of physicians with the simplicity of editing images, contrast and presentation of details, clarity of pathological status, ability to zoom, trustworthiness of the system and images’ results, ability to compare previous and new images of a patient and system’s user-friendliness in PACS was higher than traditional radiology systems. There was no significant difference between the two systems in the above-mentioned components (Table 3) (*p* > 0.061).

**Table 3:** Mean score of satisfaction with PACS versus traditional radiology

Features	Mean score of satisfaction with digital imaging system (PACS)	Mean score of satisfaction with analogue imaging (film-based)	***P***-value
Easy to edit images	4.37±0.97	2.53±1.35	0.061
Contrast or presenting image’s details	4.49±0.87	2.33±1.09	0.46
Clarity of pathological status	3.98±1.29	2.74±1.16	0.464
Ability to zoom images	4.45±0.89	2.27±1.1	0.854
System reliability	3.71±1.34	2.69±1.33	0.205
Reliability of the images’ findings	3.98±1.12	2.73±1.18	0.95
Possibility to compare patient’s previous and new images	4.36±1.13	2.97±1.4	0.514
Easy to use the system	4.05±1.29	2.57±1.42	0.084

The major weakness points of PACS for physicians included wasting time when looking up images on computer systems (*n* = 6), inability to print images and inability to access these images at other medical centers outside the hospitals such as physician’s office for follow up. Other weaknesses were increasing patients’ costs for being forced to go through the imaging process repeatedly and being exposed to x-ray again (*n* = 5), inability to use this system at the bedside of patients and increasing the time required to search images (*n* = 4). The ability to change color and edit images, especially images of the brain (*n* = 1) is one of the most important strengths of this system.

## Discussion

The results showed that most users of Picture Archiving and Communication System, are satisfied with this system. In this study, radiologists and also physicians who had higher computer literacy were more satisfied with PACS. Physicians believed that because of having different features such as editing, ability to apply different changes to the images and also the ability to compare patients’ previous and new images, this system and its findings are more reliable. Despite all advantages of PACS, several physicians believed that this system somewhat wastes their time. Currently, inability to use PACS images at other healthcare centers outside the hospital can increase patients’ costs for re-imaging and also the risk of being re-exposed to x-ray. The advantages of PACS identified in this study are discussed in the following sections.

### Optimization in work process, efficiency and quality of service

The results of the present study showed that more than half of the physicians believed using PACS improves work process, quality of care and also training. In line with this finding, Tan’s [15] study also revealed that more than two-thirds of users believed that the PACS enhances their performance and compared to the traditional system of hard copies, this system improves physicians’ performance. The findings of two other studies [7, 10] also showed improvement in the quality of healthcare services, productivity and efficiency as the results of using PACS.

### System’s ease of use

More than half of the physicians in this study believed that reviewing images with this system is easy and the PACS has met their expectations. A study by Buabbas and colleagues [10] showed that more than three-quarters of the radiologists and technologists were positive about PACS and found it user-friendly. Also, Jorwekar and colleagues [36] in their study addressed the easiness of using PACS and, consistent with our results, reported that 85% of the users believed that PACS was very easy to use for them. According to the results of this research and previous similar studies it seems that despite use of a variety of tools and menus in PACS interface, it is highly easy to learn for users and can meet users’ expectations in this regard.

### Reducing hospital-stay time

In this study, more than half of the physicians believed that PACS has no influence on reducing the patients’ length of stay at hospital. Despite these results, Al-Alawi’s study showed that about two-thirds of PACS users agreed that this system reduces patients’ hospital stay [9]. Some studies have investigated the impact of PACS on patient length of stay. The findings of these studies [37–39] showed that this system can reduce the duration of patient stay at hospital. However, a previous study [40] showed that PACS does not affect the patients’ length of stay at hospital.

Discrepancy in the findings of different studies on PACS can be due to a variety of reasons. However, one of the most important reasons may be the difference in the population of studies. The difference of the images required for patients in different wards or hospitals can present different findings. Also, users with different standpoints that are being studied can lead to different results. Leastwise in Iran the PACS is still considered a relatively new system and may be the clinical advantages of this system has not fully perceived by physicians.

### Reducing costs

According to our findings, more than half of the physicians believed that using PACS can reduce costs. PACS-related costs are divided into two categories of direct and indirect costs. Direct costs are expenses related to implementing the PACSs such as system purchase cost, maintenance, and equipment purchase. While the indirect costs include increasing patients’ hospital stay, repeating similar imaging, reducing productivity and physician’s performance for the lack of access to images and reports [41].

Regarding PACS-related costs, there are different perspectives. While some studies address the high cost of purchase, implementation, and maintenance of the PACS [42–45], other studies point out the effectiveness of this system and also consider the reduction of indirect costs as the result of PACS’s implementation [12, 46]. By reducing indirect costs, PACS and can compensate for the direct expenses inflicted on the hospital and can even lead to the reduction of general expenses [41].

Fang et al. study [47] showed that an appropriately designed PACS can save financial resources and reduce indirect costs compared to film-based imaging due to the increasing productivity of devices and technicians, providing the opportunity of online phone consultation, saving time for physicians and radiologists and decreasing the number of required personnel.

Physicians take different approaches towards PACS due to their different standpoints regarding direct and indirect costs [41]. Presumably, some physicians fail to take the reduction of indirect costs in the long-term into consideration, and therefore they believe that PACS increases costs. Despite this, indirect costs of PACS are significantly lower compared to the traditional system.

### Patients’ safety

According to 10% of physicians, because of the inability to print images or access them at other treatment centers outside the hospital such as physicians’ offices, the patients are forced to redo the radiographies, which further expose them to x-ray and finally affect patient safety. Despite the findings of the present study, Modrak and colleagues [14] in a study showed a decrease in the exposure to x-rays after implementation of PACS due to lower need to repeat radiographies. Also, other studies have shown that implementation of PACS can reduce repeated radiographies, which prevents unnecessary patient harm due to exposure to x-rays [48, 49]. This difference could be partly related to the difference information network infrastructure used. In Iran, images are only communicated within an organization and there is no connection with other organizations. In Iran, PACSs are not integrated into other health information systems such as hospital information systems. For this reason, physicians believe that patients need to repeat imaging and exposure to x-rays if they refer to other treatment centers. However, when the communication of images between health information systems used at governmental and private health care centers is established, PACS can decrease patients’ exposure to x-rays by improving access to the same image from different locations.

### PACS versus traditional radiology

The results of the present study show that most physicians believe that because the PACS has different features such as image edit, ability to apply changes to images such as contrast, clarity, and zoom, and also the ability to present details, this system is more satisfying for them compared to traditional radiology. However, there was no significant difference in the ease of use of two systems. In Abuabbas and colleagues’ [10] study, most participants suggested that the system was user-friendly. Also, Al Yafei and colleagues [50] in their study reported 90% user-friendliness for the PACS. The results of the mentioned studies are consistent with the findings of present study. However, the results of present study about comparing the ease of use in PACS and the traditional system differ somewhat from Jorwekar and colleagues’ study [36] in which system users described the PACS as being very user-friendly. Perhaps this level of difference in opinions about the easiness in using PACS compared to traditional film-based systems is related to the computer literacy level of the users and or the lake of interoperability of PACS with other health information systems. It seems that higher computer literacy of users and better interoperability of the system with other systems can increase users’ satisfaction. On the other hand, Similar studies, have shown that changing habits is difficult in the clinical setting [51] and requires strong intention, positive attitudes of clinicians, and appropriate intervention [52]. Therefore, when there is resistance to the implementation of PACS among physicians, it is suggested that an appropriate intervention should be taken to change the attitude and behavior of physicians towards the use of digital systems. Creating a positive attitude in physicians towards new systems can increase their adoption and ultimately lead to more successful implementation.

Also, another reason for the difference in opinions about the system’s user-friendliness and its ease of use could be the users’ level of involvement during the system analysis phase before the design phase. If users’ needs are properly assessed, and the system is accordingly designed, then the system would probably meet users’ satisfaction.

### Relationship between satisfaction level and demographic information

The results showed that factors such as age, experience of using PACS, and physicians’ specialty had no significant relationship with the level of satisfaction; however, physicians’ computer literacy affected the level of their satisfaction. In line with these findings, Barabbas’s study [10] showed that none of the demographic information including users’ computer literacy affect their level of satisfaction.

The reason that the relationship between satisfaction and computer literacy was not similar among studies could be self-reporting of the computer literacy. We suggest that future studies use a standard computer literacy questionnaire along with the satisfaction questionnaire to precisely investigate the relationship between these two variables.

Although the sample size in the present study was not very large, this was the first study in Iran that assessed users’ satisfaction with PACS. The physicians who took part in this study had experience in working with PACS and were interested in completing the questionnaire. This study provided an easy to review list of PACS advantages and weaknesses for the managers and policy makers of healthcare centers who are considering purchasing or implementing a PACS in their organizations. Particularly, the results suggest that in the development of these system a due attention should be paid on establishing a communications network of this system with other health information systems and with other health care centers.

## Conclusions

The results revealed that although the mean scores of physicians’ satisfaction with PACS was at a moderate to a high level, yet there are still problems in successfully implementing this system and establishing interoperability between them at different treatment centers. The results showed that PACS have not fully met all the demands of physicians and has not achieved its predetermined objectives in some healthcare centers, such as all-access from different locations. To overcome some of the existing problems, the results specifically suggest increasing the number of workstations for these systems or to use a personal digital assistant (PDA) to reduce time spent to reach a station and to facilitate providing care at patients’ bedside, user-specific training for more successful of system implementation. Also adding the printing feature to the system can be useful for sending the picture to other centers outside the hospital.

## Data Availability

The data generated and analyzed during this study are available from the corresponding author on reasonable request.

## Abbreviations

LOS: Length of stay
PACS: Picture Archiving and Communication System
PDA: Personal Digital Assistan
